# The Hypoxic Burden, Clinical Implication of a New Biomarker in the Cardiovascular Management of Sleep Apnea Patients: A Systematic Review

**DOI:** 10.31083/j.rcm2505172

**Published:** 2024-05-16

**Authors:** Carlota Coso, Esther Solano-Pérez, Sofía Romero-Peralta, María Castillo-García, Laura Silgado-Martínez, Sonia López-Monzoni, Pilar Resano-Barrio, Irene Cano-Pumarega, Manuel Sánchez-de-la-Torre, Olga Mediano

**Affiliations:** ^1^Sleep Unit, Pneumology Department, Hospital Universitario de Guadalajara, 19002 Guadalajara, Spain; ^2^Centro de Investigación Biomédica en Red de Enfermedades Respiratorias (CIBERES), 28029 Madrid, Spain; ^3^Instituto de Investigación Sanitaria de Castilla La Mancha (IDISCAM), 45071 Toledo, Spain; ^4^Sleep Research Institute, 28036 Madrid, Spain; ^5^Medicine Department, Universidad de Alcalá, 28805 Madrid, Spain; ^6^Sleep Unit, Pneumology Department, Hospital Universitario Ramón y Cajal, Instituto Ramón y Cajal de Investigación Sanitaria (IRYCIS), 28034 Madrid, Spain; ^7^Precision Medicine Group in Chronic Diseases, Respiratory Department, Hospital Universitario Arnau de Vilanova y Santa María, 5198 Lleida, Spain; ^8^Department of Nursing and Physiotherapy, Faculty of Nursing and Physiotherapy, Universidad de Lleida, IRBLleida, 25002 Lleida, Spain

**Keywords:** hypoxic burden, cardiovascular, sleep apnea, biomarkers, clinical practice

## Abstract

**Background:**

Obstructive sleep apnea (OSA) is a highly prevalent sleep-disordered breathing. It is associated with adverse co-morbidities, being the most scientific evidence of cardiovascular (CV) disease. Currently, OSA is measured through the apnea-hypopnea index (AHI), the total number of respiratory events per hour of sleep. However, different studies have questioned its utility in OSA management, highlighting the need to search for new parameters that better reflect the heterogeneity of the disease. Hypoxic burden (HB) has emerged as a novel biomarker that informs about the frequency, duration and depth of the desaturation related to the respiratory events. We conducted a systematic review in order to find publications about the heterogeneity of OSA measured by HB and its associations with future disease.

**Methods:**

Systematic review was conducted using PubMed and Web of Science. The terms “sleep apne” and “hypoxic burden” were used to look for publications from the date of inception to August 15, 2023. Inclusion criteria: articles in English published in peer-reviewed journals. Exclusion criteria: (1) not available publications; (2) duplicated articles; (3) letters, editorials, and congress communications; (4) articles not including information about HB as a specific biomarker of OSA.

**Results:**

33 studies were included. The results were classified in 2 main sections: (1) HB implication in the CV sphere: HB showed to be a better predictor of CV risk in OSA patients than traditional measures such as AHI with possible clinical management implication in OSA. (2) HB response to OSA treatment: pharmacological and nonpharmacological treatments have demonstrated to be effective in improving hypoxia measured through the HB.

**Conclusions:**

HB could be a better and more effective parameter than traditional measurements in terms of diagnosis, risk prediction and therapeutic decisions in patients with OSA. This measure could be incorporated in sleep units and could play a role in OSA management, driving the clinic to a more personalized medicine.

## 1. Introduction

Obstructive sleep apnea (OSA) is a sleep-disordered breathing (SDB) characterized by repetitive episodes of total (apnea) or partial (hypopnea) obstructions of the upper airway during sleep. These alterations in normal ventilation cause intermittent hypoxia, changes in intrathoracic pressure and sleep fragmentation, which contribute to the development of significant impairments on health [1]. It is estimated that OSA is present in nearly one billion people worldwide, with a prevalence of moderate-to-severe OSA between 3–9% in females and 10–17% in males [2, 3, 4].

Overnight, in laboratory polysomnography (PSG) is the gold standard test for the diagnosis of OSA [5]. However, PSG is a complex test that is not always available, meaning alternative diagnostic tests may be performed [5]. The American Academy of Sleep Medicine (AASM) guidelines recommend home sleep apnea tests (HSAT) for the diagnosis of adult patients with a high probability of moderate-to-severe OSA [6] and without serious comorbidities. HSAT usually measures airflow, respiratory effort, oxygen saturation (${\mathrm{SpO}}_{2}$) and heart rate. Since it has limitations because it does not include electroencephalogram, electrooculogram or electromyogram, a negative result should be considered for further investigation with PSG [1].

OSA is measured through the apnea-hypopnea index (AHI), defined as the total number of respiratory events recorded per hour of sleep and is obtained from sleep studies. The International Consensus Document considers OSA when: (1) the presence of an AHI ≥15/h, and is predominantly obstructive; (2) the presence of an AHI ≥5/h accompanied by one or more of the following factors: excessive sleepiness during the day, restless sleep, excessive fatigue and/or impaired sleep-related quality of life, not justifiable by other causes [7]. OSA classification is divided as: no OSA when AHI is less than 5 events per hour, mild OSA when AHI is between 5 and 15 events per hour, moderate OSA if AHI is between 15 and 30 events per hour, and severe OSA when AHI is greater than 30 events per hour.

The most effective treatment is continuous positive airway pressure (CPAP), which mitigates AHI and hypoxia. However, other treatments are available when CPAP is not recommended or ineffective. Among alternatives to CPAP, body weight loss should be recommended for all overweight or obese patients with OSA [8]. In a subgroup of patients, oral appliance devices and CPAP are similarly effective in terms of their impact on symptoms and quality of life [9, 10], being higher adherence to these devices. Finally, surgical and medical treatments (positional therapy, anti-inflammatory medication and hypoglossal nerve stimulation) could be considered, although they are less frequent [11].

**Fig. 1.**
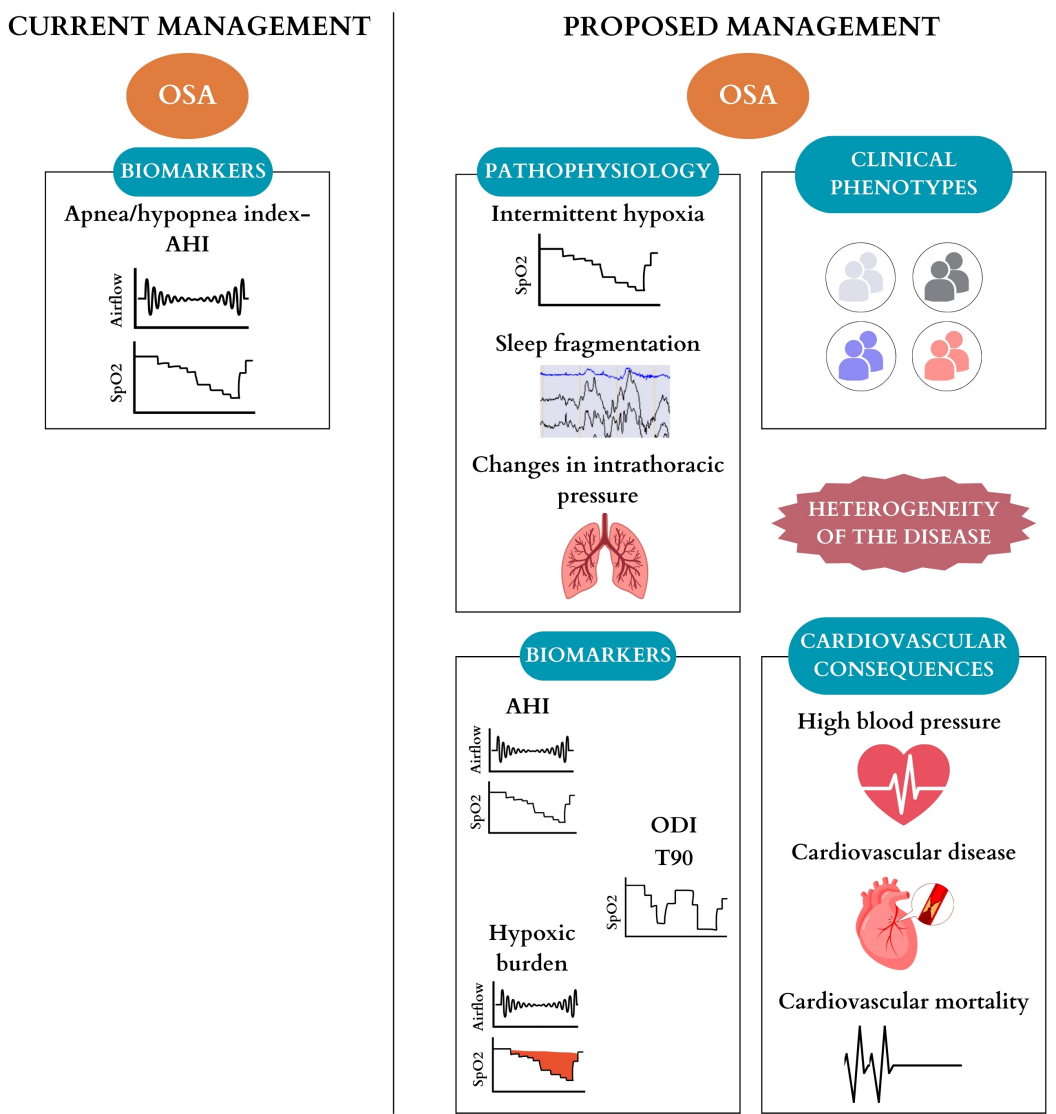
**Comparison between current management and proposed management for obstructive sleep apnea based on the heterogeneity of the disease**. Abbreviations: OSA, obstructive sleep apnea; AHI, apnea-hypopnea index; ${\mathrm{SpO}}_{2}$, oxygen saturation; ODI, oxygen desaturation index; T90, % of time with oxygen saturation below 90%.

Therefore, diagnosis and severity of OSA and treatment management are mostly determined by their AHI. However, this sole parameter does not reflect the complex heterogeneity of the disease, which is a multifactorial illness that includes different pathogenic mechanisms, clinical phenotypes [12, 13] and cardiovascular (CV) consequences (Fig. 1). An example could be that individuals with similar AHI values have different patterns of hypoxia, the main deleterious factor involved in the principal consequences of OSA [14], and distinct clinical daytime repercussions [15]. In this sense, there has been an effort to move beyond AHI for OSA clinical management. First, cluster analyses have identified groups of OSA patients with similar characteristics including symptoms, sleep disturbances and comorbidities, which may be helpful to establish prognostic factors and personalized therapeutic strategies in OSA management [12, 13, 16]. Another important approach in OSA management has been the identification of biomarkers for patients who may be at high CV risk.

Observational studies indicate that OSA is associated with adverse morbidities, being the most scientific evidence on CV disease, and that alleviation of obstructive events with CPAP may improve such CV outcomes [17]. In this CV sphere, OSA has been predominantly associated with high blood pressure (HBP) and CV mortality, but also with other CV complications such as heart failure, coronary artery disease and stroke [18]. OSA is highly prevalent in HBP patients, between 30 to 50%, a prevalence which is increased in resistant HBP (more than 85%), and OSA is the most frequent factor of secondary HBP [19, 20]. The increase in blood pressure (BP) levels and the worsening of its control, has been suggested that is slightly reverted with treatment with CPAP [21]. Besides, although moderate-to-severe OSA has been associated with increased CV mortality [18, 22], randomized clinical trials (RCT) have failed to prove the reduction of such adverse CV events with treatment with CPAP [23, 24, 25, 26] in populations with CV disease concomitant with OSA. One of the possibilities is that this lack of effect would be related to the parameter chosen (AHI) in these RCTs for OSA characterization.

Hence, different studies have questioned the utility of AHI in OSA, highlighting that it is not the best parameter for its management, including CV disease prediction. Since there is a need to search for new parameters that better reflect this heterogeneity and predict which patients would benefit most from treatment, novel biomarkers for OSA severity have been proposed. In this review, we will focus on hypoxic burden (HB), described as a measure of OSA that captures all dimensions of OSA-related desaturations, including frequency, duration and depth of the respiratory events. HB was first defined by Azarbarzin *et al. * [27] in 2019 as the total area under the respiratory event-related desaturation curve. HB is obtained by adding all individual desaturation areas and dividing it by the total sleep time, being the units of HB (%min)/h [27].

To assess whether HB has been used to evaluate OSA, we conducted a systematic review to find publications about the heterogeneity of OSA measured by HB and if it is associated with future disease.

## 2. Materials and Methods

This systematic review was conducted using PubMed and Web of Science, and based on Preferred Reporting Items for Systematic Reviews and Meta-Analysis (PRISMA) [28]. The main focus was to identify scientific knowledge of HB as a novel measurement for OSA severity, and its clinical implication as a predictor of increased CV risk.

**Fig. 2.**
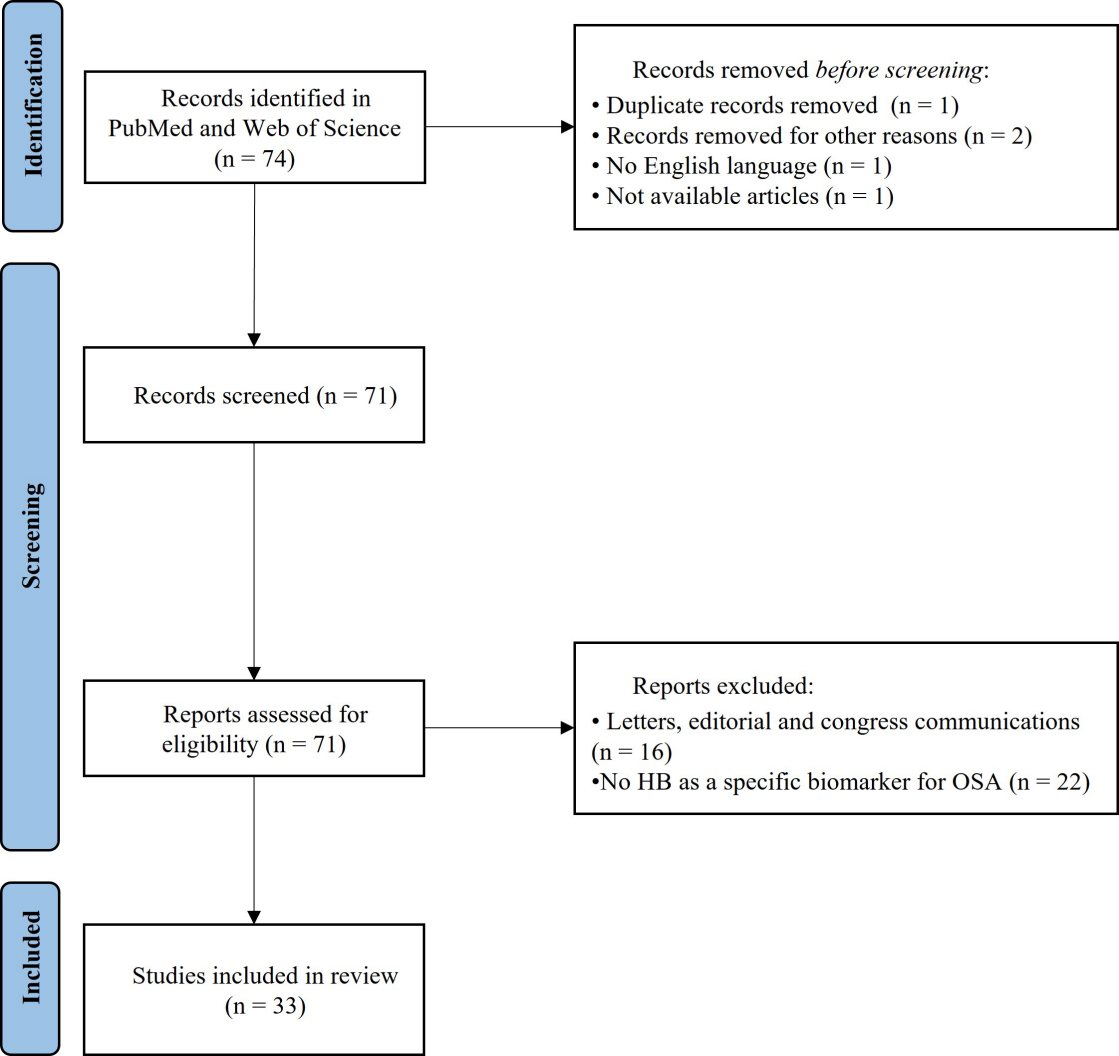
**Schematic flow diagram for the selection of reports**. Abbreviations: HB, hypoxic burden; OSA, obstructive sleep apnea.

The terms “sleep apnea” and “hypoxic burden” were used to look for all the publications from the date of inception to August 15, 2023. Inclusion criteria were: articles in English published in peer-reviewed journals. Exclusion criteria included: (1) not available publications; (2) duplicated articles; (3) letters, editorials, and congress communications; and (4) articles not including information about HB as a specific biomarker of OSA. Two independent reviewers screened the titles and abstracts of all candidates according to eligibility criteria. Data were extracted independently and included the type of study and main CV outcomes related to HB. The flow diagram is illustrated in Fig. 2.

## 3. Results

### 3.1 Study Selection

The first review of the literature identified a total of 74 studies. After the initial title and abstract research, 3 articles were excluded: 1 study was not written in the English language; 1 study was duplicated; and 1 article was not available. A total of 71 studies were screened and assessed for eligibility. After initial screening, 38 studies were removed: 16 studies were letters, editorials or communications and 22 studies did not include HB as a specific biomarker of OSA. A total of 33 studies that fulfilled the inclusion criteria and not exclusion criteria were selected for the present systematic review (Fig. 2).

### 3.2 Relationship between HB and CV Risk

The reports of the literature review were classified in 3 sections: (1) HB implication in the CV sphere, in which HB was used to better classify OSA patients according to their CV risk, studying a more consistent relationship with CV disease than AHI; (2) HB response to OSA treatment, where the efficacy of pharmacological and non-pharmacological treatments in diminishing OSA severity evaluated through HB; and (3) Other diseases, in which HB was assessed to characterize the effects of OSA in serious health conditions (corona virus disease 2019 (COVID-19) patients, intensive care unit patients and chronic kidney disease (CKD)).

The main results were found in the CV sphere (Table 1, Ref. [14, 17, 27, 29, 30, 31, 32, 33, 34, 35, 36, 37, 38, 39, 40, 41]). These reviewed studies highlight that AHI may oversimplify the complexity of the disorder and do not optimally correlate with associated CV morbidity and mortality. As OSA is a highly heterogeneous disorder, patients have different expressions of the disease, responses to treatment and susceptibility to comorbidities. Consequently, HB has emerged as a new biomarker for a better stratification of OSA related to CV risk [14, 17, 29, 30, 31, 32, 33, 34, 35] and response to therapy of OSA.

**Table 1.** **The main results of reports, including cardiovascular outcomes related to hypoxic burden as a measure of OSA**.

Report	Type of study and cohort	Main CV outcomes related to HB
Azarbarzin A. *et al*. 2019 [27]	Observational, longitudinal study	HB was defined for the first time and OSA severity quantified with this term was independently associated with CV mortality.
	MrOS and SHHS cohorts	
de Chazal P. *et al*. 2020 [30]	Review	AHI may oversimplify the complexity of OSA and poorly correlates to CV consequences. Parameters such as HB can better predict CVR and other comorbidities.
Kim JS. *et al*. 2020 [40]	Cross-sectional study	High HB was related to higher BP, specifically with increased DBP overall and SBP and DBP among non-hypertension medication users. HB was similarly associated with DBP in both NREM and REM sleep.
	MESA cohort	
Azarbarzin A. *et al*. 2020 [38]	Observational, longitudinal study	Incident HF was more strongly and consistently associated with the HB than the traditional AHI.
	MrOS and SHHS cohorts	
Azarbarzin A. *et al*. 2021 [36]	Observational, longitudinal study	HB together with ΔHR, may be useful for the identification of OSA patients with high CVR. There is an association of a high ΔHR with nonfatal and fatal CVD and all-cause mortality, moderated by the severity of OSA (higher HB).
	MESA and SHHS cohorts	
O’Donnell C. *et al*. 2021 [29]	Review	HB seems to be better correlated with the end-organ consequences of OSA than AHI.
Trzepizur W. *et al*. 2022 [37]	Observational, longitudinal study	HB and T90 were better associated with major adverse CV events than AHI and ODI. This marker could be utilized in clinical practice to identify OSA patients at higher CVR.
	Pays de la Loire Sleep cohort	
Javaheri S. and Javaheri S., 2022 [31]	Review	There may be other metrics to quantify the severity of OSA in the HF population, including HB. HB is currently under investigation, however more investigation is needed before clinical application.
Blekic N. *et al*. 2022 [35]	Review	HB is a parameter that results in a better assessment of CV OSA patients. It would be included for improving classification of OSA patients with higher future CVR.
Martinez-Garcia MA. *et al*. 2023 [32]	Review	A threshold of HB >60 %min/h is established to identify OSA with higher risk of CV morbidity and mortality. Soon, this measure could be incorporated in sleep laboratories and could play a role in clinical therapeutic decisions in patients with OSA.
Trzepizur W. *et al*. 2023 [39]	Observational, longitudinal study	HB predicted incident venous thromboembolism. Thus, patients with more severe nocturnal hypoxia are more likely to have incident venous thromboembolism.
	Pays de la Loire Sleep cohort	
Redline S. *et al. *2023 [14]	Review	HB was related with higher BP and had stronger relation with CV mortality and major incidents of CV events.
Peker Y. *et al*. 2023 [17]	Review	HB have demonstrated to be better predictors of adverse CVD outcomes and response to OSA treatment.
Solano-Pérez E. *et al*. 2023 [33]	Review	HB has not yet been assessed in the pediatric population. Evaluating HB in children with OSA could be an interesting advance to predict the risk of the disease and may improve the choice of the treatment.
Esmaeili N. *et al*. 2023 [41]	Observational, longitudinal study	The oximetry-derived HB, a novel method to quantify the HB automatically was assessed and it highly correlated with manual-scored HB and was associated with EDS, HBP and CVD mortality in a similar way as previously reported.
	SHHS cohort	
Pack AI. 2023 [34]	Review	HB and ΔHR, that may provide information on who with OSA is at most risk for CV consequences.

Table footnotes. Abbreviations: CV, cardiovascular; HB, hypoxic burden; MrOS, Osteoporotic Fractures in Men Study; SHHS, Sleep Heart Health Study; OSA, obstructive sleep apnea; AHI, apnea-hypopnea index; CVR, cardiovascular risk; MESA, Multi-Ethnic Study of Atherosclerosis; BP, blood pressure; DBP, diastolic blood pressure; SBP, systolic blood pressure; NREM, non-rapid eye movement; REM, rapid eye movement; HF, heart failure; ΔHR, heightened pulse-rate response to apneas and hypopneas; CVD, cardiovascular disease; T90, time below 90% of oxygen saturation; ODI, oxygen desaturation index; EDS, excessive daytime sleepiness; HBP, high blood pressure.

HB has been demonstrated to be a better predictor of adverse CV outcomes and response to OSA treatment. Azarbarzin A. *et al. *2019 [27], Azarbarzin A. *et al. *2021 [36] and Trzepizur W. *et al. *2022 [37] identified a better relationship between HB and major adverse CV events and mortality than AHI and other respiratory parameters such as oxygen desaturation index (ODI). In addition, time with oxygen saturation below 90% (T90) together with HB could be predictors of heart failure [38] and venous thromboembolism in different observational cohorts [31, 37, 39]. It has been also demonstrated that HB may be used for identifying patients with high values of BP, principally in diastolic BP (DBP) overall and systolic BP (SBP) and DBP among non-hypertension medication users [40]. Some authors establish a threshold of HB >60 %min/h for the identification of OSA patients with an elevated CV risk [32]. However, these results have not yet been evaluated in children, an ideal population free of established diseases and other confounding factors present in adults [33].

In summary, the intermittent hypoxia produced in OSA is better characterized by this new biomarker and has demonstrated a direct implication in the development of CV disease. Thus, HB seems to be a better predictor of CV risk in OSA patients than traditional measures such as AHI.

### 3.3 HB and the Response of OSA Treatment

A large number of studies evaluating the efficacy of treatments in OSA was found in the literature search (Table 2, Ref. [42, 43, 44, 45, 46, 47, 48, 49, 50]). These studies included HB and AHI as the OSA severity measurements, used to evaluate the efficacy of different treatments for OSA. In general, the analyzed drugs were effective in reducing HB and AHI of moderate-to-severe OSA, at least in the short term. The most utilized drug was atomoxetine, which was combined with different medicines to reduce OSA severity. In this sense, Schweitzer P*. et al*. 2023 [42] combined atomoxetine plus oxybutynin in patients with moderate pharyngeal collapsibility, reducing OSA severity in these patients. Messineo L*. et al*. 2022 [43]assessed atomoxetine and fesoterodine, obtaining better results in a post hoc analysis including participants characterized by milder collapsibility. In another study, Messineo L. *et al*. 2023 [44] showed that atomoxetine in combination with dronabinol could reduce OSA severity, but more studies are needed as it produced numerous side effects. Finally, Corser B. *et al*. 2023 [45] found that atomoxetine plus the hypnotic trazodone significantly reduced AHI and HB, probably driven by an increase in pharyngeal muscle activity during the events.

**Table 2.** **Main results of reports including the response of OSA treatment measured through HB**.

Report	Type of study	Main outcomes related to HB
Mullins A. *et al*. 2021 [47]	Observational	The HB during slow wave sleep was significantly reduced during CPAP withdrawal with supplemental oxygen compared to CPAP withdrawal without oxygen. Sleep fragmentation was maintained but HB was significantly reduced.
Schweitzer P. *et al*. 2023 [42]	RCT	Atomoxetine plus oxybutynin significantly improved OSA severity measures such as AHI and HB in patients with moderate pharyngeal collapsibility. This effect on HB was not significantly different from that of atomoxetine alone.
Messineo L. *et al*. 2022 [43]	RCT	Atomoxetine and fesoterodine had no effect on AHI, but led to a trend for HB reduction (not statistically significant), accompanied by signs of improved pharyngeal collapsibility. Post hoc analysis revealed that participants with milder collapsibility exhibited a significant reduction of NREM HB.
Messineo L. *et al*. 2022 [48]	RCT	In a subgroup of patients in which there was an increase of the arousal threshold, pimavanserin exhibited a decrease in AHI and HB.
Rosenberg R. *et al*. 2022 [49]	RCT	Patients treated with high and low doses of antimuscarinic aroxybutynin had a statistically significant and clinically meaningful difference from placebo in HB.
Huang W. *et al*. 2023 [46]	RCT	The combination of positional therapy with oral appliance on positional OSA was better than either alone in improving AHI and HB. This could implicate a reverse the course of the disease and lowers the risk of complications by alleviating hypoxemia as reflected by the HB.
Corser B. *et al*. 2023 [45]	RCT	Atomoxetine plus the hypnotic trazodone significantly reduced AHI and HB, probably driven by an increase in pharyngeal muscle activity during the events. However, atomoxetine plus another hypnotic called lemborexant had smaller effects.
Messineo L. *et al*. 2023 [44]	RCT	Atomoxetine in combination with dronabinol might be useful to reduce OSA severity (AHI and HB) in those who could tolerate the combination. However, given the numerous side effects, these results warrant further validation in larger trials.
Berger M. *et al*. 2023 [50]	RCT	Administration of oxybutynin plus reboxetine did not improve AHI, but they improved oxygen desaturation and HB. However, the combination reduced sleep efficiency and sleep quality.

Abbreviations: HB, hypoxic burden; CPAP, continuous positive airway pressure; RCT, randomized clinical trial; AHI, apnea and hypopnea index; OSA, obstructive sleep apnea; NREM, non rapid eye movement.

On the other hand, different treatments apart from drugs were assessed. Positional therapy in combination with oral appliances improved AHI and HB of the patients included in the study [46], suggesting that they could lower the risk of complications by alleviating hypoxemia as reflected by the HB. Finally, HB was evaluated in OSA patients during CPAP withdrawal, in which hypoxia was significantly reduced when supplemental oxygen was delivered in slow-wave sleep [47].

Briefly, different OSA treatments, including pharmacological and nonpharmacological, have been demonstrated to be effective in improving hypoxia measured through the HB, pointing out that it could be an adequate marker for evaluating OSA as it better characterizes the immediate consequences produced in this illness.

### 3.4 HB Related with Different Comorbidities

Finally, few studies evaluated the implication of HB in other morbidities. OSA produces intermittent episodes of hypoxemia and reoxygenation that predispose patients with OSA to worsen health conditions, contributing to the development of different disorders [51].

On the one hand, these nocturnal desaturations during sleep may worsen the symptoms of acute respiratory failure in severe COVID-19. Celejewska-Wójcik N. *et al*. 2023 [52] showed that the number of respiratory events, HB and the number of desaturations were related to the requirement for more advanced respiratory support.

Undiagnosed SDB is common in the intensive care unit (ICU), as suggested by Bucklin A. *et al*. 2023 [53]. They described for the first time the HB of ICU patients, being 17% of patients with a HB ≥30 %min/h, and 8% HB ≥50 %min/h. They also showed that there was a low correlation between HB and more general hypoxia indices, demonstrating that apnea-specific hypoxia is not well measured with standard indices.

In the end, given that OSA exerts much of its deleterious effects on the kidney through hypoxemia, Jackson C. *et al*. 2021 [54] observed a significantly higher moderate-to-severe CKD prevalence in the highest vs. lowest HB group of the Multi-Ethnic Study of Atherosclerosis (MESA) cohort. This significant prevalence was maintained in the participants in the highest group of HB plus AHI ≥5/h.

In conclusion, specific HB may be able to better characterize the effects of OSA compared to current measurements of frequency such as the AHI in serious health conditions (COVID-19 severe patients, and ICU patients) and other diseases like CKD.

## 4. Discussion

There is enough evidence to affirm that AHI by itself is outdated to characterize OSA, focusing on the remodeling of OSA management in developing novel markers that consider symptoms and comorbid illnesses. Currently, classifying OSA severity based on the number of observed events oversimplifies the pathophysiology of the disease. Different variables such as HB collect more information about the respiratory event.

The strengths of HB comprise, firstly, that this parameter describes the frequency, duration and depth of the desaturation associated with the obstructive episodes, which presents a higher relationship with associated consequences and morbidities than classical metrics.

Secondly, as intermittent hypoxia is considered the principal damaging factor for developing CV disease, HB is an innovative measurement specific to OSA and has recently been used to characterize it. Until now, conventional measures including ODI and T90 are better related to CV outcomes than AHI [55], but they share similar limitations. On the one hand, ODI only measures frequency but not the duration or depth of the events, and there is not a consensus on the percentage drop to be considered, being 3% or 4% depending on the study criteria. T90 measures persistent hypoxemia but not intermittent hypoxia that is not specific to OSA and that may be correlated with chronic airway diseases. Finally, similar to AHI, both metrics do not take into account the basal saturation of the patients. Hence, the HB could provide additional clinical information rather than classic values. OSA categorization in severity groups employing HB could differentiate patients with high HB and low HB, at the same time that this population is grouped according to their associated CV risk. More RCTs are needed to establish the cut-off point of OSA patients with high HB, although the first reports have shown a limit of 60 %min/h [32].

Thirdly, in the same way as the classical methods, HB can also be obtained from simplified methods, since it is calculated from the data acquired from the flow and saturation signals. Moreover, it can be calculated from automatic analyses of the sleep studies, facilitating the work of clinicians and accelerating the diagnosis. A recent publication [41] has assessed the relationship between manual-scored HB and automatically-scored HB, showing a high correlation between them and with adverse CV outcomes in a similar way as reported. Thus, it could be used to contribute to reducing the underdiagnosis observed in this disease.

Fourthly, it has been proposed that HB could identify the patients that would benefit from treatment and represent an important target for improving CV risk. Despite the beneficial effects of CPAP in CV risk reduction (improvements in endothelial dysfunction, blood pressure and first signs of atherosclerosis) in OSA patients, there is controversy about the role of CPAP in the prevention of adverse CV events. In the three principal RCTs [23, 24, 26] evaluating CPAP in CV outcomes, there were no significant associations between CPAP and adverse CV events or death. A possible explanation could be the selection of the patients included in such studies, as they had previous CV illnesses, suggesting a possible decreased beneficial effect in secondary CV risk prevention. The heterogeneous pathophysiology of OSA stands out that a personalized approach accounting for specific endotypes of patients with OSA is necessary and worthwhile. If we properly determine the different OSA phenotypes that exist, using HB among other variables, we could be able to identify patients who could benefit from CPAP as secondary prevention. Thus, more studies are needed to evaluate the possible HB utilization for detecting those specific profiles of patients. In this line, a recent study from Pinilla L. *et al*. 2023 [56] reported that in a population with acute coronary syndrome and OSA, the group with higher HB had a significant reduction in the incidence of CV events when they were treated with CPAP. These results demonstrate the possibility of implementing HB as a measure of OSA severity, differentiating patients in which CPAP could have a long-term protective effect on CV prognosis.

Lastly, the different responses to treatment and different prognoses could be evaluated through HB. It has been reviewed that different drugs and other non-pharmacological treatments alternative to CPAP lower HB but not AHI in all cases. A possible explanation could be that they decrease the duration and depth of the desaturation but not the number of respiratory events. Consequently, HB would help in the selection of the treatment in terms of reducing the risk of future disease.

However, the limitations of HB need to be commented on. The main disadvantage is the inability to differentiate long and more superficial desaturations from short but deep ones. For example, an HB of 60 (%min)/h could correspond to 10 min of 6% desaturation or 20 min of 3% desaturation per hour of sleep. Besides, it cannot detect the arousals associated with the respiratory event, which may underestimate the diagnosis. Another weakness is the incapacity to distinguish apneas from hypopneas. Finally, there is no consensus on how to use it in practice or to calculate it to ensure that everyone obtains the same HB. Nevertheless, these limitations could be complemented with other parameters, symptoms and other illnesses, creating a score that includes various aspects of the disease.

In summary, HB has more advantages compared to current management, which justifies studying its feasibility to be implemented in clinical practice.

## 5. Conclusions

HB has been used in numerous studies to assess OSA, emphasizing its correlation with the CV domain. It appears to surpass traditional measurements in terms of diagnosis based on comorbidities and making clinical therapeutic decisions for OSA patients. Integrating this measure into sleep units could enhance OSA management and contribute to a shift towards more personalized medicine in clinical practice. Furthermore, this approach not only addresses the immediate concerns associated with OSA but also recognizes and manages potential CV implications.

## Abbreviations

OSA, obstructive sleep apnea; SDB, sleep-disordered breathing; PSG, polysomnography; AASM, American Academy of Sleep Medicine; HSAT, home sleep apnea test; ${\mathrm{SpO}}_{2}$, oxygen saturation; AHI, apnea-hypopnea index; CPAP, continuous positive airway pressure; CV, cardiovascular; HBP, high blood pressure; BP, blood pressure; RCT, randomized clinical trials; HB, hypoxic burden; PRISMA, Preferred Reporting Items for Systematic Reviews and Meta-analysis; CKD, chronic kidney disease; ODI, oxygen desaturation index; T90, time with oxygen saturation below 90%; DBP, diastolic blood pressure; SBP, systolic blood pressure; ICU, intensive care unit; MESA, Multi-Ethnic Study of Atherosclerosis.

## Supplementary Material

Supplementary material associated with this article can be found, in the online version, at https://doi.org/10.31083/j.rcm2505172.
